# Fingolimod induces neuroprotective factors in human astrocytes

**DOI:** 10.1186/s12974-015-0393-6

**Published:** 2015-09-30

**Authors:** Franziska S. Hoffmann, Johann Hofereiter, Heike Rübsamen, Johannes Melms, Sigrid Schwarz, Hans Faber, Peter Weber, Benno Pütz, Verena Loleit, Frank Weber, Reinhard Hohlfeld, Edgar Meinl, Markus Krumbholz

**Affiliations:** Institute of Clinical Neuroimmunology, Ludwig Maximilian University, 81377 Munich, Germany; German Center for Neurodegenerative Diseases (DZNE) and Technical University, 81377 Munich, Germany; Max Planck Institute of Psychiatry, 80804 Munich, Germany; Center of Neurology and Hertie Institute for Clinical Brain Research, University of Tübingen, Tübingen, Germany; Munich Cluster for Systems Neurology (SyNergy), Munich, Germany

**Keywords:** Fingolimod, Astrocyte, Neuroprotection, Leukemia inhibitory factor, Interleukin 11, Heparin-binding EGF-like growth factor, B-cell activating factor of the TNF family/TNFSF13b, CXCL10/IP10, MX1, OAS2

## Abstract

**Background:**

Fingolimod (FTY720) is the first sphingosine-1-phosphate (S1P) receptor modulator approved for the treatment of multiple sclerosis. The phosphorylated active metabolite FTY720-phosphate (FTY-P) interferes with lymphocyte trafficking. In addition, it accumulates in the CNS and reduces brain atrophy in multiple sclerosis (MS), and neuroprotective effects are hypothesized.

**Methods:**

Human primary astrocytes as well as human astrocytoma cells were stimulated with FTY-P or S1P. We analyzed gene expression by a genome-wide microarray and validated induced candidate genes by quantitative PCR (qPCR) and ELISA. To identify the S1P-receptor subtypes involved, we applied a membrane-impermeable S1P analog (dihydro-S1P), receptor subtype specific agonists and antagonists, as well as RNAi silencing.

**Results:**

FTY-P induced leukemia inhibitory factor (*LIF*), interleukin 11 (*IL11*), and heparin-binding EGF-like growth factor (*HBEGF*) mRNA, as well as secretion of LIF and IL11 protein. In order to mimic an inflammatory milieu as observed in active MS lesions, we combined FTY-P application with tumor necrosis factor (TNF). In the presence of this key inflammatory cytokine, FTY-P synergistically induced *LIF*, *HBEGF*, and *IL11* mRNA, as well as secretion of LIF and IL11 protein. TNF itself induced inflammatory, B-cell promoting, and antiviral factors (*CXCL10*, *BAFF*, *MX1*, *and OAS2*). Their induction was blocked by FTY-P. After continuous exposure of cells to FTY-P or S1P for up to 7 days, the extent of induction of neurotrophic factors and the suppression of TNF-induced inflammatory genes declined but was still detectable. The induction of neurotrophic factors was mediated via surface S1P receptors 1 (S1PR1) and 3 (S1PR3).

**Conclusions:**

We identified effects of FTY-P on astrocytes, namely induction of neurotrophic mediators (*LIF*, *HBEGF*, and *IL11*) and inhibition of TNF-induced inflammatory genes (*CXCL10*, *BAFF*, *MX1*, and *OAS2*). This supports the view that a part of the effects of fingolimod may be mediated via astrocytes.

**Electronic supplementary material:**

The online version of this article (doi:10.1186/s12974-015-0393-6) contains supplementary material, which is available to authorized users.

## Background

Fingolimod (FTY720) reduces relapses, disability progression, and brain atrophy in patients with relapsing-remitting multiple sclerosis (MS) [1, 2]. FTY720 is a synthetic analog to natural sphingosine. Both are rapidly phosphorylated by sphingosine kinase 1/2 (SPK1/2) in blood and tissue to the active compounds FTY720-phosphate (FTY-P) and sphingosine-1-phosphate (S1P). Inactivation involves reversible dephosphorylation by two phosphatases, SGPP1 and SGPP2, and degradation by a lyase, SGPL1. S1P binds to five S1P receptors (S1PR1-5), but also direct intracellular signaling has been described [3, 4]. FTY-P is a ligand for four of these receptors, S1PR1 and S1PR3-5 [5]. S1P receptors are G protein coupled receptors, which are internalized after ligand binding.

Both FTY-P and S1P are agonists in short-term. While after S1P binding the receptor is recycled back to the surface within minutes [6], this is impaired by the alkyl side chain of FTY-P [7], resulting in receptor downregulation and functional antagonism of FTY-P in lymphocytes after prolonged exposure [8]. This leads to blood lymphopenia, since CCR7+ lymphocytes are no longer guided from lymphatic tissue to the bloodstream by the S1P gradient [8]. Lymphocyte trapping in lymphatic organs is considered a main mode of action of FTY720 therapy in MS. However, details of signaling and receptor kinetics may differ in other cell types [7, 9–11].

In addition to its effects in lymphoid organs, FTY720 has, owing to its lipophilic nature, access to and accumulates in the CNS [12], where S1P receptors are expressed on all brain resident cells [12]. Astrocytes are the most abundant cell type within the CNS and predominantly express S1PR1 and S1PR3 [9, 13]. Astrocytes maintain CNS homeostasis under physiologic conditions, and astrocyte dysfunction perpetuates CNS diseases [14]. In particular, astrocytes are able to produce neuroprotective messengers and can play a beneficial role supporting oligodendrocyte and axonal regeneration [15] but can also secrete inflammatory cytokines and actively promote CNS inflammation via adaptive and innate mechanisms [16]. In MS, astrocytes produce increased amounts of BAFF and CXCL10, fostering B cells and recruiting monocytes and T cells [17, 18].

A decisive role of neurodegeneration in MS pathogenesis is increasingly being recognized [19]. While focal inflammatory activity as the immunological correlate of relapses can efficiently be reduced by several available compounds, preventing loss of axons and brain atrophy is still a therapeutic challenge. Brain atrophy is an independent predictor of disability in patients with relapsing-remitting MS [20, 21] but was reduced during FTY720 treatment by half [1, 22]. In conjunction with in vitro effects of FTY-P on brain resident cells and an experimental autoimmune encephalomyelitis (EAE) model with potential involvement of CNS S1P receptors in the therapeutic effect of FTY720 [23], the idea of neuroprotection by FTY720 has been formulated. Neuroprotection may be the consequence of reduced number and activity of immune cells entering the CNS, or of CNS-intrinsic effects such as direct protection of neuronal and oligodendrocytic integrity and function, or of beneficial effects mediated indirectly via other CNS resident cells. For example, fingolimod was shown to block NO-production induced by S1P or inflammatory cytokines, resulting in inhibition of astrocyte-mediated neurodegeneration [24].

In this study, we focused on potential neuroprotective mechanisms of FTY-P exerted via astrocytic S1P receptors. We observed that FTY-P induced neuroprotective factors (leukemia inhibitory factor (*LIF*), interleukin 11 (*IL11*), and heparin-binding EGF-like growth factor (*HBEGF*)), and suppressed the tumor necrosis factor (TNF)-induced inflammatory cytokines *BAFF* and *CXCL10* (aka interferon inducible protein-10, IP10) as well as antiviral proteins like 2′-5′-oligoadenylate synthetase 2 (*OAS2*) and myxovirus resistance 1 (*MX1*). We suggest that FTY720 modulates the microenvironment in the brain via effects on astrocytes.

## Methods

### Cell culture

Human astrocytes of embryonic origin, devoid of microglial cells or macrophages [25], as well as U373 astrocytoma cells were cultured in Dulbecco’s modified Eagle’s medium (DMEM) (Life Technologies, Darmstadt, Germany) containing 10 % FCS (Biochrom/Merck, Berlin, Germany) and 100 U/ml penicillin and 100 μg/ml streptomycin (Gibco, Invitrogen). Cells were plated at a concentration of 100,000 cells/ml. Before stimulation, all cells were switched to serum-free medium (Panserin 401, Pan-Biotech, Aidenbach, Germany).

All experiments with fetal human neural progenitor cells derived from striatal brain area were approved by the ethics committee of the University of Leipzig and the Technical University of Munich, Germany, in accordance with all state and federal guidelines. Human neural striatal progenitor cells (hstrNPCs) were generated from CNS tissue of spontaneously aborted human fetuses (gestational weeks 10, 12, and 14) with mother’s consent as described. Briefly, the human fetal tissue of gestational weeks 10, 12, and 14 was washed with sterile Hanks’ balanced salt solution (HBSS) and dissected into mesencephalic and non-mesencephalic primary tissue samples. The tissues were mechanically separated into small pieces, incubated in 0.1 mg/ml papain solution (Roche), supplemented with 10 μg/ml DNase (Roche) for 30 min at 37 °C, then washed three times with HBSS followed by an incubation with 50 μg/ml antipain solution (Roche) for 30 min at 37 °C. After three further washing steps, samples were homogenized 20 times by gentle trituration. Propagation of the cell suspension was performed in poly-L-ornithine (Sigma-Aldrich) and fibronectin (Chemicon) coated cell culture flasks. The expansion medium was based on DMEM/Ham’s F12 mixture (PAA, Laboratories) supplemented with 2 % B27 (Invitrogen), hrEGF, and hrFGF-2 (20 ng/ml, Peprotech). Growth factors were supplemented every other day. Long-term expansion of the cells (>6 months) was enabled in reduced atmospheric oxygen (2–3 %). For passaging, cell detachment was induced by Accutase™ (PAA Laboratories) for 30 min at 37 °C at a confluency of 80–100 %. For differentiation, cells were plated onto pre-coated cell culture dishes. After the cells reached 80–100 % confluency, the medium was exchanged to Neurobasal medium (Invitrogen, Germany) containing additives such as B-27 minus-AO supplement (Invitrogen), Forskolin (Sigma-Aldrich), and db cyclic AMP (Sigma-Aldrich). The cells were differentiated for 7 days.

### Compounds

We used the following reagents: S1P (SL-140; 100 nM, 1 μM; Enzo Life Biosciences), dihydro-S1P (SL-143; 100 nM, 1 μM; Enzo Life Biosciences), FTY-P (B-0721; 1 μM; Echelon Biosciences/Mobitec) (all dissolved in methanol), W146 (3602; 1, 10 μM; Tocris; in NaOH), TY52156 (5328, 1, 10 μM; Tocris; in ethanol), SEW-2871 (H1109D; 1, 10 μM; Biomol; in DMSO), CYM5541 (4897; 1, 10 μM; Tocris; in DMSO), and TNF (R&D Systems, in PBS). Concentrations of FTY-P and S1P were chosen according to pilot experiments for optimal effects on established S1P induced genes, not for equimolar concentrations of S1P and FTY-P. In all experiments, vehicle controls with the respective concentration of solvents were included to control for removal of autocrine trophic factors and cellular stress.

### RNA, cDNA, and qPCR

RNA was isolated using the Qiagen RNeasy Mini Kit including DNase digestion (Qiagen, Hilden, Germany) according to the manufacturer’s instructions. cDNA was prepared using the High Capacity cDNA Archive Kit (Applied Biosystems, Darmstadt, Germany). Quantitative PCR (qPCR) was performed on the ABI 7900HT Fast Real-Time PCR thermocycler (Applied Biosystems) using the qPCR core kit and uracil N-glycosylase (both from Eurogentec, Cologne, Germany). For all reactions, the annealing temperature was 60 °C. We used the following primer/probes: LIF, IL11, HBEGF, S1PR1-5, OAS2, SPHK1, SPHK2, SGPL1, SGPP1, LIFR, EGFR, IL11RA (TaqMan Gene Expression Assays, Applied Biosystems), BAFF [18], MX1 [26], and CXCL10 [27]. Cyclophilin A (peptidyl-prolyl isomerase A (PPIA)), glyceraldehyde 3-phosphate dehydrogenase (GAPDH), and beta-actin (all Applied Biosystems) were used as housekeeping genes. To validate the house-keeping genes, we stimulated human primary astrocytes or human U373 astrocytoma cells with FTY-P, S1P, and TNF and determined expression of PPIA, GAPDH, and beta-actin by quantitative PCR using equal amounts of RNA. We found no relevant regulation of the analyzed house-keeping genes by any of the applied stimuli, and expression levels for PPIA and GAPDH were most stable (Additional file 1: Figure S1). Therefore, PPIA and GAPDH were used in the subsequent experiments.

### Microarray

Screening for FTY-P induced genes was performed on the Illumina gene expression microarray platform (Illumina, Munich, Germany). RNA concentration, purity, and quality were checked on the Nanophotometer (Implen, Munich, Germany) and the Agilent 2100 Bioanalyzer (Agilent Technologies, Palo Alto, CA, USA). All samples had a RNA integrity number ≥9.8. RNA was amplified and labeled using the TotalPrep RNA Amplification Kit (Ambion, Houston, TX, USA) and hybridized onto human 12v3 whole genome gene expression arrays following the manufacturer’s instructions (Illumina).

Fluorescence intensity values were extracted and computed to *beadsummary* data by a BeadArray Reader (Illumina) using the company’s standard parameters. No additional background correction beyond that done by Illumina’s standard protocol was performed. The manufacturer’s built-in controls were analyzed including hybridization controls and sample-dependent parameters. Illumina’s recommendations for quality control were fulfilled. Data was loaded into R [28] using package *beadarray* for all subsequent calculations.

Eleven out of 48,803 probes listed in the annotation (0.02 %) were not technically sampled in all cRNA preparations and thus excluded from further analysis (KCNRG, PDZRN3, HS.575197, LOC648364, INDO, C7ORF27, RHOBTB1, CMIP, ZNF57, TMEM80, TMPRSS7).

Probe filtering aimed at keeping only array probes showing fluorescence levels above background. Background was defined at the median of all array probes for each individual microarray. Array probes that did not pass the threshold on any microarray were removed. Microarray data normalization was performed using the function *vsn* (*variance stabilizing normalization*) from the Bioconductor [29, 30] package vsn [31].

Differential gene expression analysis was done using the package *limma*. Significantly regulated genes were ranked using an empirical Bayes method (implementation *eBayes* from package *limma*) that uses information from the ensemble of all samples to estimate the sample variance for each gene. This approach aims at stabilizing the statistical analysis, especially for small array numbers. Correction for multiple testing was done using the false discovery rate (FDR) approach by Benjamini and Hochberg.

### ELISA

Supernatants for ELISA were harvested 8–16 h after the last stimulation. Enzyme-linked immunosorbent assay (ELISA) of cell culture supernatants was performed on Maxisorp 96-well plates (Nunc, Wiesbaden, Germany) using DuoSet ELISA kits for CXCL10, IL11, HBEGF (all R&D systems, Wiesbaden-Nordenstadt, Germany) and LIF (Bender Med Systems, Vienna, Austria) according to the manufacturer’s instructions.

### siRNA

Silencer® Select Validated siRNAs against S1PR1 and S1PR3 as well as a control siRNA were purchased from Ambion/Life Technologies. Sequences are listed in Additional file 2: Table S1. siRNAs were transfected at a concentration of 2 nM using Lipofectamine RNAimax (Life Technologies) following the manufacturer’s instructions. Twenty-four hours after siRNA transfection, cells were stimulated with FTY-P as indicated. Eight hours later, supernatant was harvested and ELISA was performed. Knock-down was validated by quantitative PCR.

### NFκB-reporter assay

U373 cells were cotransfected with a firefly luciferase reporter plasmid and the internal control CMV Renilla luciferase plasmid. Twenty-four hours later, cells were treated with increasing concentrations of FTY-P or vehicle control. One hour later, TNF-α was added as indicated, and 8 h later, cells were lysed with passive lysis buffer (Promega, Mannheim, Germany). Reporter gene activity was determined using firefly luciferase substrate (Biozym, Hamburg, Germany) and Renilla luciferase substrate (Promega), respectively.

### Statistics

Statistics and plotting was done with GraphPad Prism (GraphPad Software, La Jolla, USA) and R [28] by parametric and non-parametric tests as appropriate (two-sample tests or the respective one-sample tests if samples were tested against normalized control samples). Tests are indicated in each figure legend. *P* values of *p* < 0.05 (*), *p* < 0.01 (**), *p* < 0.001 (***) and *p* < 0.0001 (****) were considered statistically significant.

## Results

### FTY-P induces neuroprotective factors

**Fig. 1:**
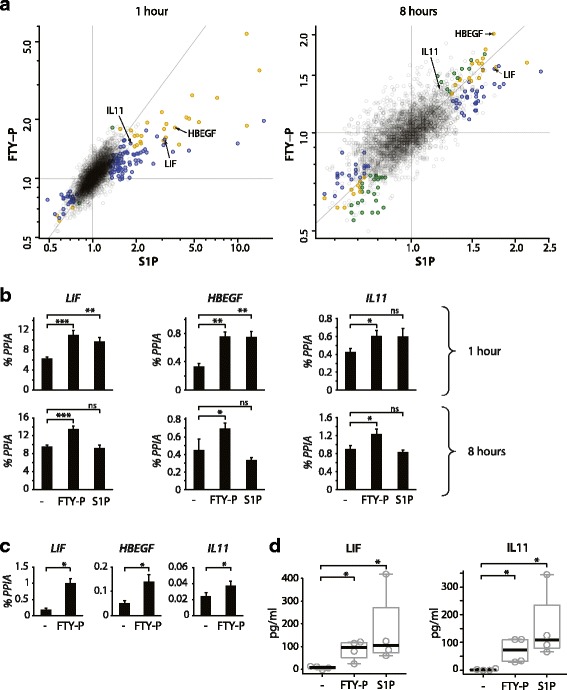
FTY-P induces expression of neurotrophic factors. **a** Human primary astrocytes were stimulated with FTY-P (1 μM), S1P (0.05 μM), or vehicle control for 1 and 8 h. Each experimental group consisted of quadruplicate (FTY-P, S1P) or triplicate (vehicle controls) wells per time point. Gene expression was determined using the Illumina microarray platform. The fold-change of expression by FTY-P and S1P is shown for each gene. Significance of regulation is indicated by the color (*green*: significant regulation by FTY-P; *blue*: significant regulation by S1P; *orange*: significant regulation by both; *transparent gray*: no significant regulation; two-tailed *t* tests adjusted for multiple comparison by the method by Benjamini & Hochberg). See also Additional file 2: Table S1 for details. Human primary astrocytes (**b**) and human U373 astrocytoma cells (**c**) were stimulated as in **a. b** *LIF*, *IL11*, and *HBEGF* expression in human primary astrocytes was determined after 1 and 8 h by qPCR (mean ± SEM of six independent biological replicates; paired two-tailed *t* tests). **c** *LIF*, *IL11*, and *HBEGF* expression in human U373 astrocytoma cells was determined after 8 h by qPCR (mean ± SEM of seven independent biological replicates; two-tailed Wilcoxon signed rank test). **d** LIF and IL11 protein secretion from U373 astrocytoma cells was determined by ELISA 8 h after stimulation (*boxplots* indicate median and first/third quartile of four independent biological replicates, with whiskers extending to outliers up to 1.5 × interquartile range; one-tailed Wilcoxon rank sum test)

To identify effects of FTY-P on astrocytes, we stimulated primary human astrocytes with FTY-P or S1P for 1 and 8 h and analyzed gene expression on the Illumina microarray platform. Since in cell culture, sphingosine and FTY720 are not efficiently phosphorylated like in blood and brain, we used the pre-phosphorylated compounds (S1P, FTY-P). We identified a panel of genes induced by FTY-P and S1P (Additional file 3: Table S2). Fold-changes of individual mRNAs correlated between stimulation with FTY-P and S1P (*p* < 2.2κ10^−16^ for both 1 and 8 h, no gene with significant opposite regulation for FTY-P vs. S1P), consistent with S1P receptor agonistic signaling for both ligands (Fig. 1). *LIF*, *HBEGF*, and *IL11* were among the most upregulated genes after 1 and/or 8 h of stimulation (Fig. 1a). The induction of *LIF*, *IL11*, and *HBEGF* mRNA in human astrocytes by FTY-P was confirmed in independent experiments on primary astrocytes (Fig. 1b) and human astrocytoma cells by qPCR (Fig. 1c). ELISA of supernatants demonstrated the induction on the protein level for LIF and IL11 (Fig. 1d), whereas we were unable to detect HBEGF in any cell culture supernatant, possibly due to its sticky properties. In order to determine whether these putative neurotrophic factors could affect neuronal survival, we determined the expression of their receptors on in vitro-differentiated human neural progenitor cells derived from fetal striatal brain area. We detected high expression of receptors for LIF (LIFR) and HBEGF (EGFR). The IL11 receptor IL11RA was also expressed, however at a lower level (Additional file 4: Figure S2).

### FTY-P interacts with TNF signaling

**Fig. 2:**
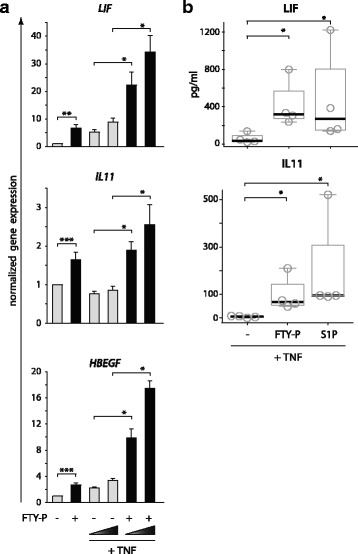
FTY-P induces neurotrophic factor also in the presence of TNF. **a** Human U373 astrocytoma cells were treated with FTY-P (1 μM) and 1 h later with different concentrations of TNF (0.005 and 0.125 μg/ml). Expression of *LIF*, *HBEGF*, and *IL11* was determined 8 h later by qPCR (values normalized to *PPIA* and the untreated control samples; mean ± SEM of seven independent experiments; two-tailed Wilcoxon signed rank test. **b** Human U373 astrocytoma cells were treated with FTY-P (1 μM) or S1P (0.1 μM), and 1 h later with TNF (0.125 μg/ml). LIF and IL11 were analyzed by ELISA after 8 h of culture (*boxplots* indicate median and first/third quartile of four independent biological replicates, with whiskers extending to outliers up to 1.5 × interquartile range; one-tailed Wilcoxon rank sum test)

We asked how emerging inflammation in the CNS might interact with FTY-P-mediated effects on astrocytes. We modeled this in vitro by pretreatment of astrocytes with FTY-P and subsequent stimulation with TNF (Fig. 2). The induction of *LIF*, *HBEGF*, and *IL11* mRNA by FTY-P was evident also in the presence of TNF, and the induction of *LIF* and *HBEGF* was even increased by TNF in a dose-dependent manner (Fig. 2a). Also, protein secretion of LIF and IL11 was induced in the presence of TNF by FTY-P (Fig. 2b). It was shown previously that S1P and TNF signaling are interconnected, as TRAF2 (TNFR-associated factor 2, which is part of the signaling complex of TNFR1) activates SPHK1 and thereby increases S1P. S1P, on the other hand, is an important factor in TNF signaling and canonical NFκB activation [4]. To further elaborate the interaction between FTY-P and TNF, we analyzed expression of *SPHK1*, along with the other FTY-P metabolizing enzymes *SPHK2*, *SGLP1*, and *SGPP1*. We detected no regulation of *SPHK2*, *SGLP1*, and *SGPP1* expression by FTY-P or TNF (Additional file 5: Figure S3A). In contrast, *SPHK1* expression was synergistically induced by FTY-P or S1P in combination with TNF (Additional file 5: Figure S3A). Further, we asked whether FTY-P has an impact on TNF-mediated NFκB activation. Using a luciferase-based reporter assay, we did not detect a major influence of FTY-P on NFκB activation (Additional file 5: Figure S3B). This is in line with previous work where S1P alone did not activate NFκB in A7 cells [4] and NFκB was activated by S1PR2, which is not targeted by FTY-P, in non-astrocytic cells [32]. S1P and FTY-P did not alter TNFR1 and TNFR2 gene expressions (Additional file 5: Figure S3C).

In summary, we provide further data on potential interaction points between TNF and S1P receptor signaling and demonstrate that regulation of TNF receptor mRNA, NFκB activation, and the synergistically induced *SPHK1* is unlikely to mediate the synergistic induction of neurotrophic factors by TNF and FTY-P.

**Fig. 3:**
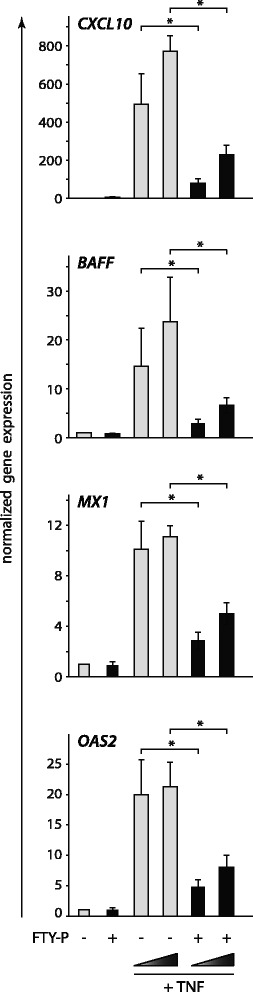
FTY-P blocks TNF-induced expression of proinflammatory and antiviral factors. Human U373 astrocytoma cells were treated with FTY-P (1 μM) and 1 h later with different concentrations of TNF (0.005; 0.125 μg/ml). Expression of *CXCL10*, *BAFF*, *MX1*, and *OAS2* was determined 8 h later by qPCR (values normalized to the untreated control samples; mean ± SEM of seven independent biological replicates; two-tailed Wilcoxon signed rank test)

**Fig. 4:**
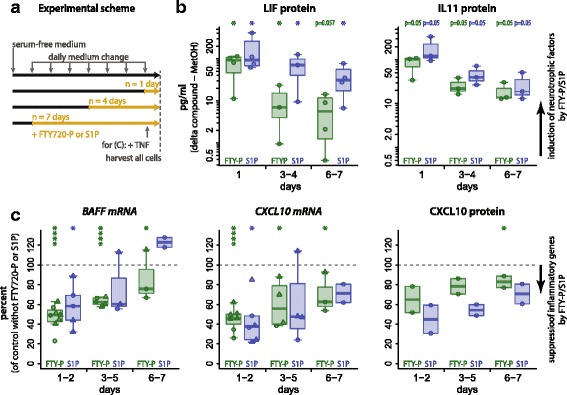
FTY-P effects are detectable also after long-term exposure. **a** For experiments shown in **b** and **c**, cells were switched to serum-free medium before the experiment. Serum-free cell culture medium was then replaced daily for up to 7 days. For the last *n* days (*orange period*), it contained additional FTY-P or S1P (*n* is displayed on the *X* axis in **b** and **c**). Thus, the total duration of serum-free cell culture was equal for all conditions per experiment. **b** U373 astrocytoma cells were treated with FTY-P (1 μM) or S1P (0.1 μM) for the last 1 and 6 days (one experiment) or the last 1, 4 and 7 days (3 experiments). Supernatants were harvested 8–16 h after the last stimulation. IL11 and LIF were measured by ELISA. Values from the vehicle control (averages for LIF 7.6 pg/ml, IL11: 2.7 pg/ml) were subtracted from the FTY-P and S1P stimulated cells. *Boxplots* indicate median and first/third quartile, with whiskers extending to outliers up to 1.5 × interquartile range; one-tailed Wilcoxon rank sum test. **c** Human astrocytes of embryonic origin (*triangles*) or U373 astrocytoma cells (*circles*) were stimulated with FTY-P (1 μM) or S1P (0.1 or 1 μM) for the last *n* days. Multiple data points per time point represent independent biological replicates. One hour after the last FTY-P application, TNF (0.025 μg/ml) was added. Cell lysates were harvested 8–16 h after TNF application. BAFF mRNA, CXCL10 mRNA, and CXCL10 protein were determined by qPCR and ELISA. Values of FTY-P and S1P treated samples are displayed normalized to the samples without FTY-P and S1P (i.e. TNF only = 100 %). *Boxplots* indicate median and first/third quartile, with whiskers extending to outliers up to 1.5 × interquartile range; one-sample *t* tests

Next, we aimed to identify further genes modulated by FTY-P pretreatment in the context of inflammation. Using a TaqMan PCR low density array, we found that the proinflammatory genes CXCL10 (IP10) and B cell activator of the TNF family (BAFF), as well as the antiviral genes MX1 and OAS2 were induced by TNF in astrocytes and that this induction was blocked by FTY-P (data not shown). Subsequent qPCR experiments confirmed that pretreatment with FTY-P blocked the induction of these inflammatory genes (Figs. 3 and 4c).

### Effects persist after long-term application of FTY-P

Since in long-term, FTY-P exerts functional antagonistic effects on lymphocyte migration, we asked whether FTY-P stimulation of astrocytic cells occurs only for short-term or also after repeated stimulation in long term. We were able to maintain U373 cells and primary human astrocytes at good viability at serum-free conditions for up to 1 week. To model continuous exposure of CNS astrocytes in cell culture, we daily added FTY-P or S1P in new serum-free medium to the cells for up to 1 week (Fig. 4a). We observed that the extent of induction of LIF and IL11 mRNA (data not shown) and protein secretion (Fig. 4b), as well as the reduction of TNF-induced cytokines (Fig. 4c), was less pronounced after prolonged exposure with FTY-P compared to a short stimulation but still present after 1 week (FTY-P for 6–7 days: *p* < 0.057 (trend) for LIF, *p* < 0.05 for all other factors). Of note, not only the effect of FTY-P but also that of S1P declined (Fig. 4).

### Neurotrophic mediators are induced via membrane-bound S1P receptors

**Fig. 5:**
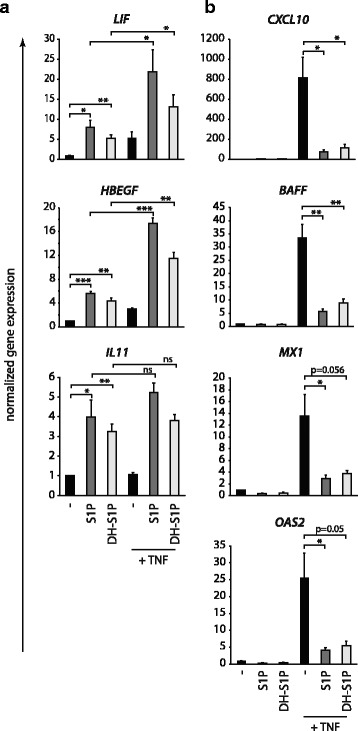
Induction of *LIF*, *HBEGF*, and *IL11*, as well as suppression of *CXCL10*, *BAFF*, *MX1* and *OAS2* is mediated via membrane receptors and not via direct intracellular signaling of S1P. Human U373 astrocytoma cells were treated with S1P (1 μM; can cross the cell membrane) or DH-S1P (1 μM; cannot cross the cell membrane) and 1 h later with TNF (0.025 μg/ml). Eight hours later, expression of **a** *LIF*, *HBEGF*, *IL11*, **b** *CXCL10*, *BAFF*, *MX1*, and *OAS2* was determined by qPCR (values normalized to *PPIA* and the untreated control samples; mean ± SEM of five independent biological replicates; one-sample two-tailed *t* test for comparison with the unstimulated cells (normalized to a value of 1), two-sample two-tailed paired *t* test for comparison between other groups). Both S1P and the non-membrane-permeable derivate DH-S1P resulted in the induction of neurotrophic factors and suppression of inflammatory genes

In addition to canonical signaling of S1P via the G protein coupled membrane-bound S1P receptors, direct intracellular effects independent of these membrane receptors have been described [4, 33]. In order to test whether induction of *LIF*, *HBEGF*, and *IL11* is mediated by surface S1P receptors, we applied the synthetic analog dihydro-S1P (DH-S1P), which—in contrast to S1P—cannot cross the plasma membrane [34]. Like S1P, also DH-S1P in equimolar concentrations induced *LIF*, *IL11*, and *HBEGF*, both in the absence and presence of TNF (Fig. 5a). The slightly lower induction by DH-S1P might be explained by the lower affinity of DH-S1P vs. S1P for S1PR1 [35]. Likewise, DH-S1P blocked the TNF-induced expression of CXCL10, BAFF, MX1, and OAS2 to a similar extent as S1P (Fig. 5b). We therefore conclude that direct intracellular signaling irrespective of membrane receptors is not the major pathway for these effects. The unphosphorylated forms, sphingosine and FTY720, did not induce any of the tested genes (data not shown).

### S1PR1 and S1PR3 mediate effects of FTY-P on astrocytes

**Fig. 6:**
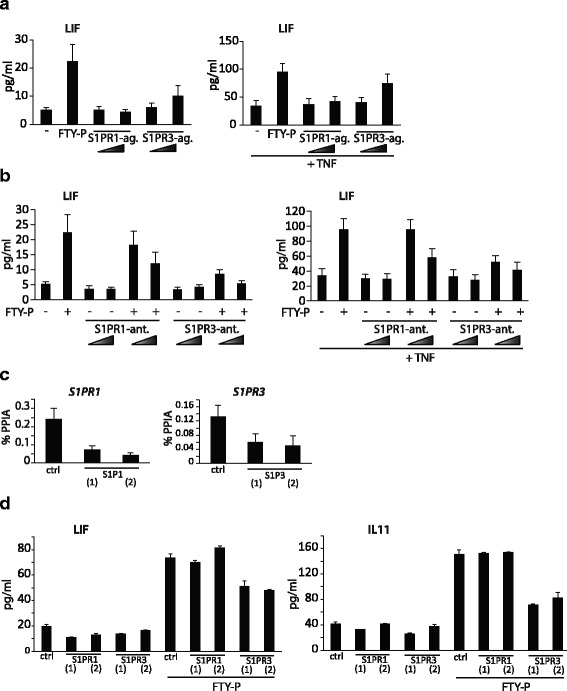
Neurotrophic factors are induced by FTY-P via S1P receptor types 1 and 3. **a** Human U373 astrocytoma cells were treated with FTY-P (1 μM) or S1P-receptor 1 (S1PR1) specific (SEW-2871, 1 and 10 μM), or S1P-receptor 3 (S1PR3) specific (CYM 5541, 1 and 10 μM), agonists and then left untreated (*left panel*) or stimulated with TNF (0.025 μg/ml) 1 h later (*right panel*). Supernatants were harvested 16 h later, and LIF production was detected by ELISA; mean ± SEM of three independent biological replicates. **b** Human U373 astrocytoma cells were pretreated with S1PR1 specific (W146, 1 and 10 μM), or S1PR3 specific (TY52156, 1 and 10 μM) antagonists, stimulated with FTY-P (1 μM), and then left untreated (*left panel*) or stimulated with TNF (0.025 μg/ml) 1 h later (*right panel*). Supernatants were harvested 16 h later, and LIF production was detected by ELISA; mean ± SEM of three independent biological replicates. **c**, **d** Human U373 astrocytoma cells were transfected with a control siRNA or two different siRNAs (2 nM) targeting S1PR1 and S1PR3, respectively, using Lipofectamine RNAimax. **c** Knock-down was validated by quantitative PCR; mean ± SEM of four independent biological replicates. **d** Twenty-four hours after siRNA transfection, cells were stimulated with FTY-P (1 μM). Eight hours later, supernatants were harvested, and LIF (*left panel*) and IL11 (*right panel*) production was determined by ELISA; representative experiment of four independent biological replicates

Both primary human astrocytes and U373 astrocytoma cells expressed predominantly S1PR1 and S1PR3 (Additional file 6: Figure S4), consistent with the literature [9, 13]. To determine which receptor is primarily involved in mediating the induction of neurotrophic factors by FTY-P, we followed different approaches. First, we applied S1PR1 (SEW-2871) and S1PR3 (CYM5541) specific *agonists* and compared induction of LIF and IL11 to the induction observed after stimulation with FTY-P. Here, we found that only the S1PR3 specific agonist induced LIF (Fig. 6a, left panel). The S1PR3 agonist also showed synergistic effects with TNF comparable to FTY-P (Fig. 6a, right panel). Second, we analyzed the effects of S1PR1 (W146) and S1PR3 (TY52156) specific *antagonists*. Blockage of both S1PR1 and S1PR3 signaling resulted in a dose-dependent reduction of *LIF* expression, with S1P3 blocking being more effective (Fig. 6b, left panel). This effect was also observed in the presence of TNF (Fig. 6b, right panel). Third, we applied *RNAi silencing* using two different siRNAs targeting S1PR1 and S1PR3, respectively. We validated knock-down by qPCR for S1PR1 and S1PR3 (Fig. 6c). Only knock-down of S1PR3 resulted in a reduction of LIF (Fig. 6d, left panel) and IL11 (Fig. 6d, right panel), while S1PR1 knock-down did not show any effect.

In summary, using different approaches (specific agonists, specific antagonists, and RNAi-mediated gene knock-down), we aimed to dissect the receptors involved the induction of neurotrophic factors and in blocking inflammatory cytokines in astrocytes. We conclude from these experiments that both S1PR1 and S1PR3 ligations may confer these effects. We cannot exclude a heterogeneous response of the cultured cells, since we measured the response of the cultures and not of single cells.

## Conclusions

### Induction of neuroprotective mediators by FTY-P

We observed that FTY-P induces *LIF*, *IL11*, and *HBEGF* gene expressions and LIF and IL11 protein secretions in human astrocytes, both in absence and presence of the inflammatory cytokine TNF. Neuroprotective effects have been attributed to these proteins: LIF protects neuronal precursor cells in the substantia nigra in a murine Parkinson’s disease model in vivo [36]. Ischemic preconditioning in the retina is mediated via LIF receptor in vivo [37]. While high LIF concentrations increase the number of MBP+ cells in spinal cord explants, but not myelination/differentiation [38], myelination is enhanced in vitro by LIF with an optimum at *low* concentrations [39] in the order of magnitude as observed in our model after 1 week of continuous stimulation, suggesting that the amount of LIF produced by FTY-P stimulation is biologically effective. *IL11*, which belongs to the *IL6* family like *LIF* and *CNTF* [40], promotes survival and maturation of oligodendrocytes and myelin formation in rodent CNS cultures [41, 42] and enhances survival of oligodendrocytes and neurons in an EAE model [42]. In addition, IL11 exerts immunoregulatory effects in rodent EAE [42]. HBEGF has been shown to restore neurogenesis in neuronal degenerative disorders and in ischemia induced brain injury [43]. Furthermore, it can enhance the survival of dopaminergic neurons [44]. Hence, a neurotrophic capacity of these proteins has been established previously and their induction by FTY-P reported in this study suggests a potential role of FTY-P in neuroprotection. We observed that the endogenous ligand S1P has principally similar effects inducing neuroprotective mediators in astrocytes. Thus, one may speculate that the pharmacological agent may enhance an already existing endogenous feature of the S1P system in human astrocytes.

### Suppression of TNF-induced inflammatory cytokines by FTY-P

In addition, FTY-P suppressed TNF-induced expression of inflammatory cytokines (BAFF, CXCL10), which could likely contribute to its beneficial effect on inflammation. BAFF and CXCL10 are key mediators in neuroinflammation: BAFF expression is elevated in MS lesions to levels observed in lymphatic organs [18]. Staining localized BAFF to astrocytes and activated astrocytes can produce greater amounts of bioactive BAFF per cell than activated macrophages, suggesting that BAFF derived from astrocytes is quantitatively meaningful [18]. Therefore, BAFF is thought to be a relevant part of the B-cell fostering environment and to perpetuate the immune response observed in the CNS of patients with MS [45]. In addition, BAFF was reported to bind to rodent neurons via BAFF-R [46] and NOGO-R [47]. Functional consequences in humans deserve further elaboration. CXCL10 binds to CXCR3 expressed i.a. on many mononuclear immune cell types. When present in the CNS, it recruits inflammatory mononuclear cells to the CNS and contributes to EAE pathogenesis [48]. TNF is a prototypic inflammatory cytokine produced by immune cells and CNS resident cells in the context of inflammation. TNF is present in active MS lesions [49], and TNF CSF concentrations correlate with disease progression in MS [50]. Soluble TNF can exert toxic effects directly via death-domain containing TNFR1 on oligodendrocytes and neurons [51, 52] and indirectly via astrocytes, which might then amplify inflammatory signals. Plain TNF antagonists, however, abolish also protective signaling for neurons and oligodendrocytes via TNFR2 and provoke or exacerbate MS [53, 54]. Suppressing proinflammatory cytokines produced by astrocytes downstream of TNF circumvents the risks associated with total TNF blockade [53]. Therefore, this is a perspective to limit further inflammation without the risk of reducing TNFR2-mediated protective signals. This constitutes a complementary approach to inhibitors selective for TNFR1 or soluble TNF [55, 56]. In contrast to the inhibition of TNF-induced CXCL10 demonstrated here, a recent study did not observe inhibition of CXCL10 induced by IL1β in astrocytes, but FTY-P was applied only at a much lower concentration of 0.1 μM [57].

### Suppression of antiviral proteins by FTY-P

Adverse events in fingolimod treated patients include upper respiratory tract infections. Neurotropic herpes virus infection and reactivation occurred more frequently in the fingolimod-treated patients [22]. MX1 and OAS2 are antiviral proteins that play an important role in the type I interferon-mediated response against a broad range of viral infections [58–60]. Blocking of the antiviral mediators MX1 and OAS2 might extend the spectrum of immunosuppressive effects of FTY-P beyond impaired CCR7+ lymphocyte egress from secondary lymphatic organs and impaired antigen shuttling in the spleen marginal zone [61].

### Effects persist during continuous stimulation with FTY-P

Since the main mode of action of FTY-P known in lymphocytes is receptor downmodulation and functional antagonism, we elaborated whether our findings are detectable also during continuous stimulation for up to 1 week. Albeit at a lower level, all effects persisted. Furthermore, there was no fundamental difference between S1P and FTY-P stimulation, as would be expected in the case of functional antagonistic effects by FTY-P as opposed to agonistic effects by S1P. This suggests that receptor agonistic signaling is present in long term, possibly because receptor downmodulation may be incomplete and partially compensated by persistent signaling after internalization [7, 9]. In line with this concept, also repeated application of FTY-P for 3 days results in sustained inhibition of intracellular calcium release by IL1β in human astrocytes. In fact, profound differences regarding S1PR1 expression, regulation, and signaling between lymphocytes and other cell types have been described: lymphocyte recirculation was suggested to be “exquisitely sensitive” [10] to receptor downmodulation as opposed to other cell types. Reasons might include lower surface expression of S1P1R [10] and a higher threshold of occupancy needed for cellular activation [11] in lymphocytes. In addition, e.g., endothelial cells, but not lymphocytes, have a substantial intracellular reserve of S1P1R, allowing for more continuous signaling [11]. Taken together, the sustained induction of neurotrophic factors and suppression of inflammatory genes in astrocytes by repeated application of FTY-P is in line with different signaling and receptor kinetics in different cell types.

### Receptors involved

Using a ligand that cannot cross the cell membrane (DH-S1P) [34], we excluded the possibility that induction of neurotrophic factors and reduction of inflammatory cytokines is mediated primarily by direct intracellular effects [4, 11], which would not be subjected to receptor downmodulation.

However, we demonstrated an involvement of surface S1PR3 and to a lesser extent S1PR1. The involvement of the S1PR1 is in line with an EAE model utilizing several knockout approaches, where the therapeutic effect of FTY720 was reported to be linked to S1P1R on astrocytes [23]. The important role of S1PR1 in EAE was further strengthened by the finding that defective phosphorylation of S1PR1 exacerbated TH17-mediated autoimmune neuroinflammation [62]. Our data indicate that also S1PR3 contributes to induction of neurotrophic factors by FTY-P.

### Summary

Taken together, we observed that FTY-P induced neurotrophic factors and blocked inflammatory cytokines in astrocytes. These findings open the possibility that a part of the beneficial effects of FTY720 could be mediated via astrocytes.

## Additional files

Additional file 1: Figure S1. Validation of different house-keeping genes for quantitative PCR in human astrocytes or U373 astrocytoma cells. Human astrocytes (A) or human U373 astrocytoma cells (B, C) were stimulated with the indicated amounts of FTY-P or S1P, followed by stimulation with TNF, when indicated. Eight hours later, cell lysates were harvested and expression of the housekeeping genes GAPDH, beta-actin, and PPIA (cyclophilin) was determined by quantitative PCR (mean ± SEM of two (A) and three (B, C) independent biological replicates). Additional file 2: Table S1. siRNA sequences. Sequences of the sense strand of siRNAs targeting S1P1 and S1P3 are listed. All siRNAs are Silencer® Select Validated siRNAs (Life Technologies). Additional file 3: Table S2. Human primary astrocytes, stimulated with FTY-P (left) or S1P (right) for 1 or 8 h and analyzed on an Illumina microarray. Shown are genes with significant regulation with at least one of the compounds (adjusted *p* value < 0.05, in bold print) at 1 h (upper table) and 8 h (lower table). The list is sorted for fold-changes by FTY-P. Additional file 4: Figure S2. Expression of *LIFR*, *EGFR*, and *IL11RA* on human fetal neural progenitor cells. Fetal Striatal brain cells were differentiated for 7 days (hstrNPCs) and analyzed by qPCR. For comparison, human primary astrocytes (HA) are shown (mean ± SD of two technical replicates). Additional file 5: Figure S3. Analysis of possible interactions between FTY-P and TNF. (A) Human U373 astrocytoma cells were stimulated with FTY-P (1 μM) or S1P (1 μM), followed after 1 h by stimulation with TNF (0.025 μg/ml) when indicated. Eight hours later, cell lysates were harvested and expression of *SPHK1*, *SPHK2*, *SGPL1*, and *SGPP1* was determined by quantitative PCR (mean ± SEM of five independent biological replicates; two-tailed paired *t* tests). (B) Human U373 astrocytoma cells were transfected with a luciferase-based NFκB-reporter and stimulated with FTY-P (1 μM) and TNF (0.025 μg/ml) 1 h later. Eight hours later, NFκB-activation was determined (mean ± SEM of combined data of 8–11 independent biological replicates). (C) FTY-P and S1P do not increase TNF receptor expression. Human primary astrocytes were treated with FTY-P, S1P, or vehicle control for 1 or 8 h (see Fig. 1, same microarray experiment). Normalized gene expression for TNFRSF1A (the main receptor for soluble TNF) and TNFRSF1B is displayed on the *Y* axis. Boxplots indicate median and first/third quartile, with whiskers extending to outliers up to 1.5 × interquartile range. Additional file 6: Figure S4. Human primary astrocytes and U373 astrocytoma cells mainly express S1P receptor types 1 and 3. Expression of *S1PR1*-*5* was determined in human primary astrocytes (A) and human U373 astrocytoma cells (B) by qPCR (mean ± SD of two technical replicates).

## Abbreviations

DH-S1P: dihydro-sphingosine-1-phosphate
FTY-P: FTY720-phosphate
HBEGF: heparin-binding EGF-like growth factor
hstrNPC: human striatal neuronal precursor cells
IL11: interleukin 11
LIF: leukemia inhibitory factor
MX1: myxovirus resistance 1
OAS2: 2′-5′-oligoadenylate synthetase 2
S1P: sphingosine-1-phosphate
TNF: tumor necrosis factor
