# Supplementary figures and images for: Fingolimod induces neuroprotective factors in human astrocytes
Source: J Neuroinflammation. 2015 Sep 30;12:184. doi: 10.1186/s12974-015-0393-6 (PMC4589103; doi:10.1186/s12974-015-0393-6)

**A**

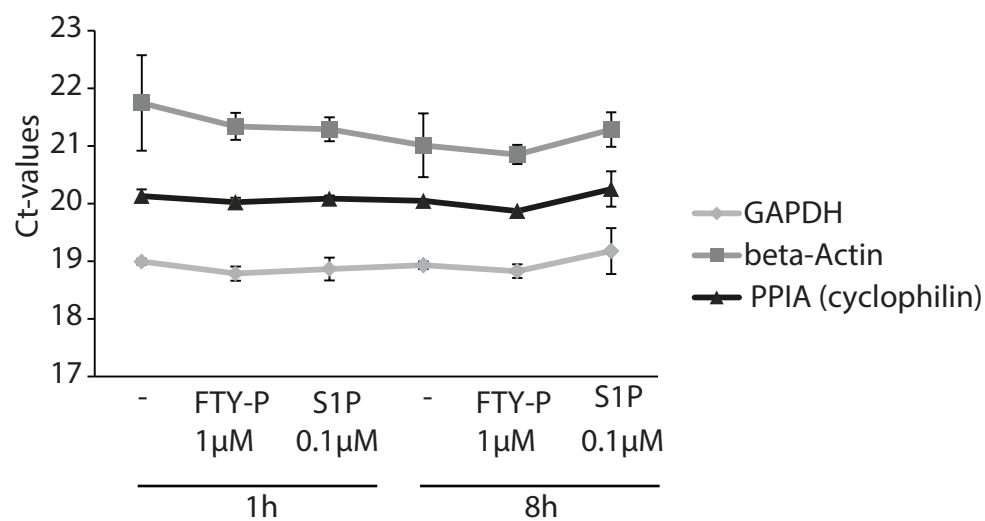

**B**

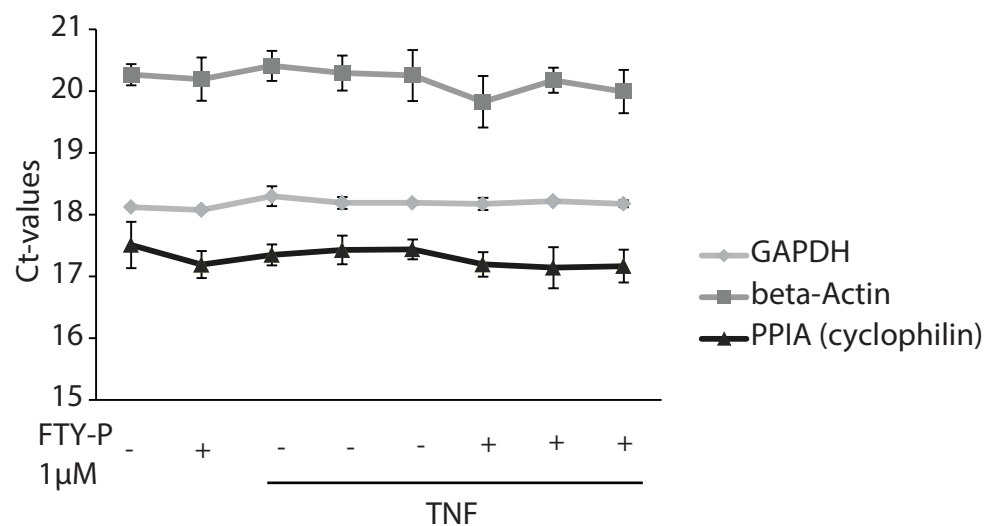

**C**

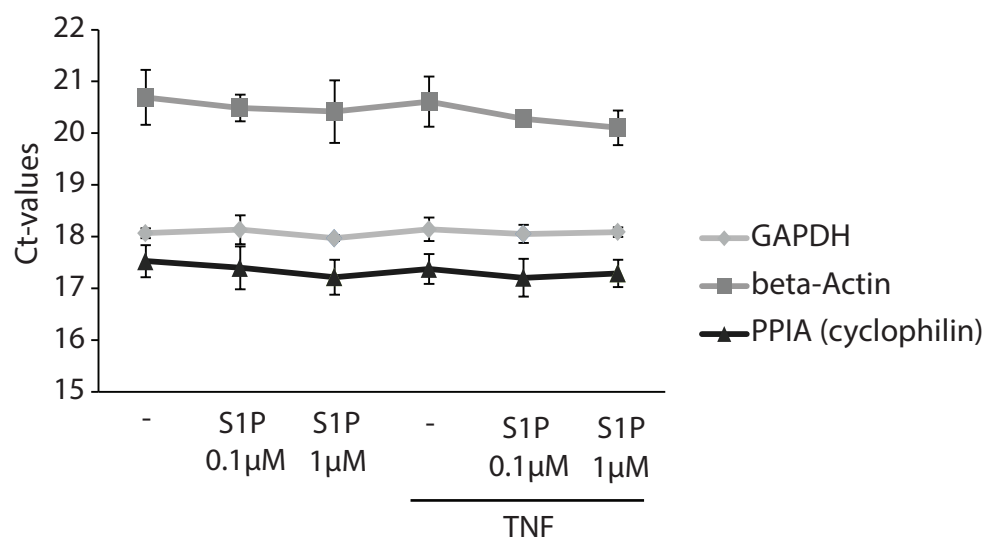

Supplement: Additional file 1: Figure S1. — Validation of different house-keeping genes for quantitative PCR in human astrocytes or U373 astrocytoma cells. Human astrocytes (A) or human U373 astrocytoma cells (B, C) were stimulated with the indicated amounts of FTY-P or S1P, followed by stimulation with TNF, when indicated. Eight hours later, cell lysates were harvested and expression of the housekeeping genes GAPDH, beta-actin, and PPIA (cyclophilin) was determined by quantitative PCR (mean ± SEM of two (A) and three (B, C) independent biological replicates). [file 12974_2015_393_MOESM1_ESM.pdf]

Suppl. Fig. 2

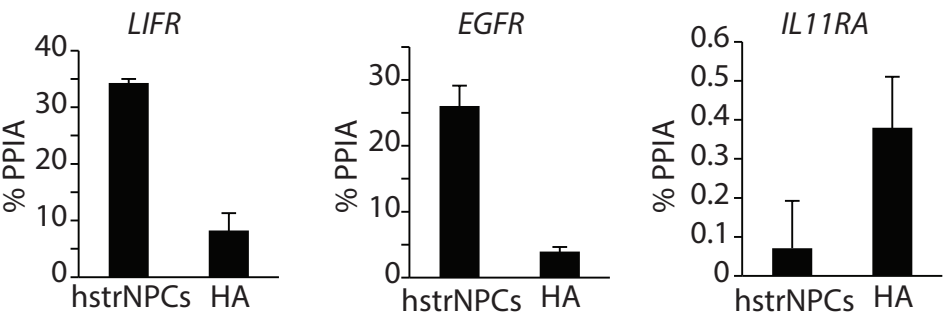

Supplement: Additional file 4: Figure S2. — Expression of LIFR, EGFR, and IL11RA on human fetal neural progenitor cells. Fetal Striatal brain cells were differentiated for 7 days (hstrNPCs) and analyzed by qPCR. For comparison, human primary astrocytes (HA) are shown (mean ± SD of two technical replicates). [file 12974_2015_393_MOESM4_ESM.pdf]

**A**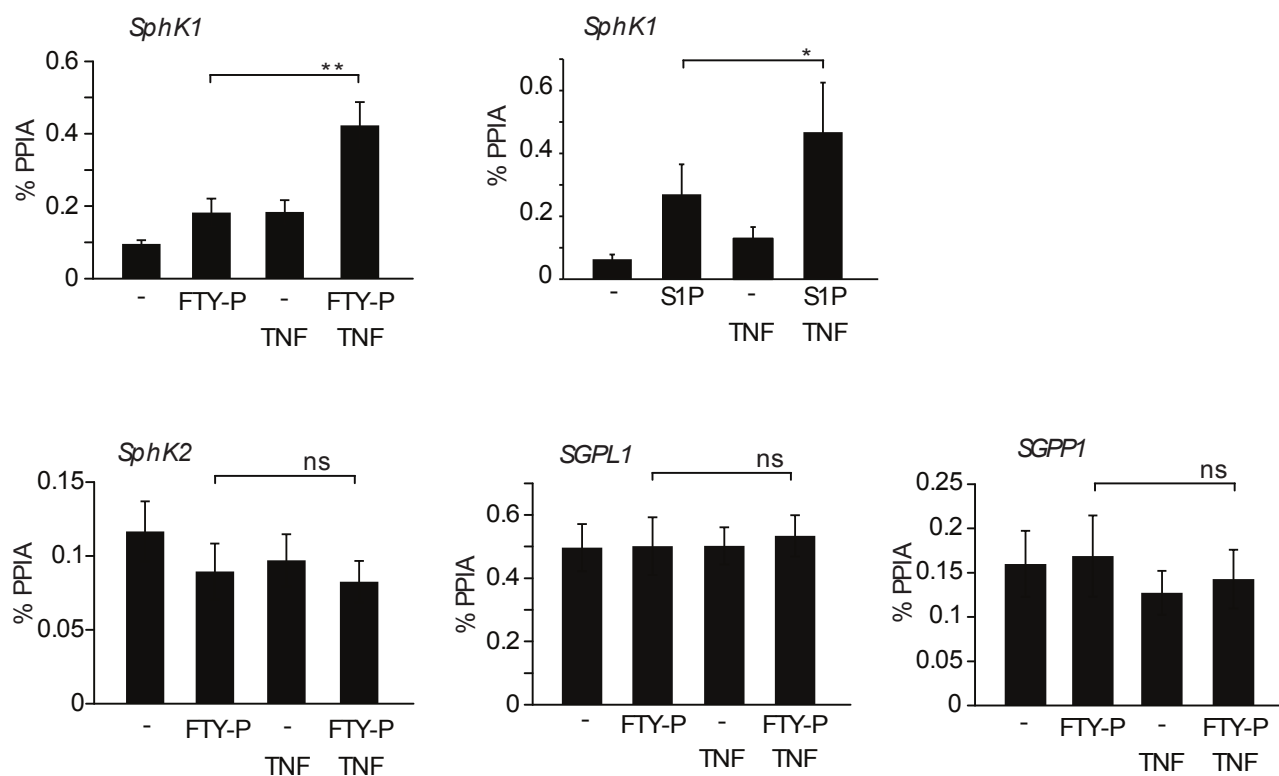**B**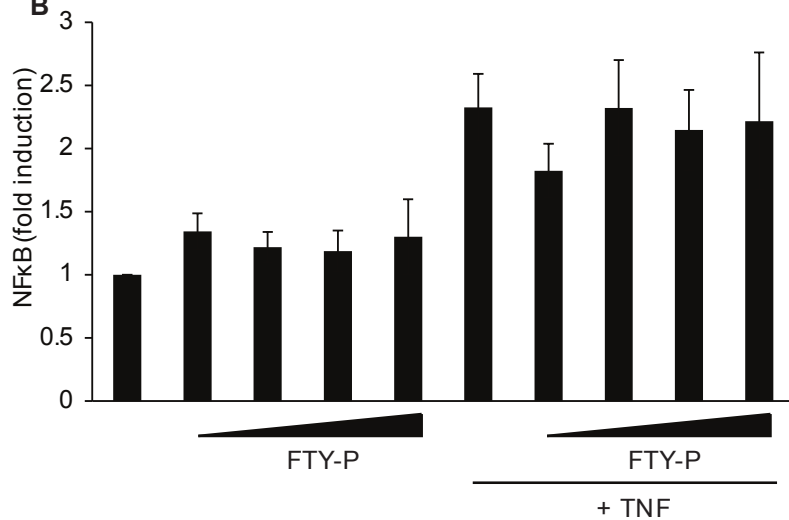**C**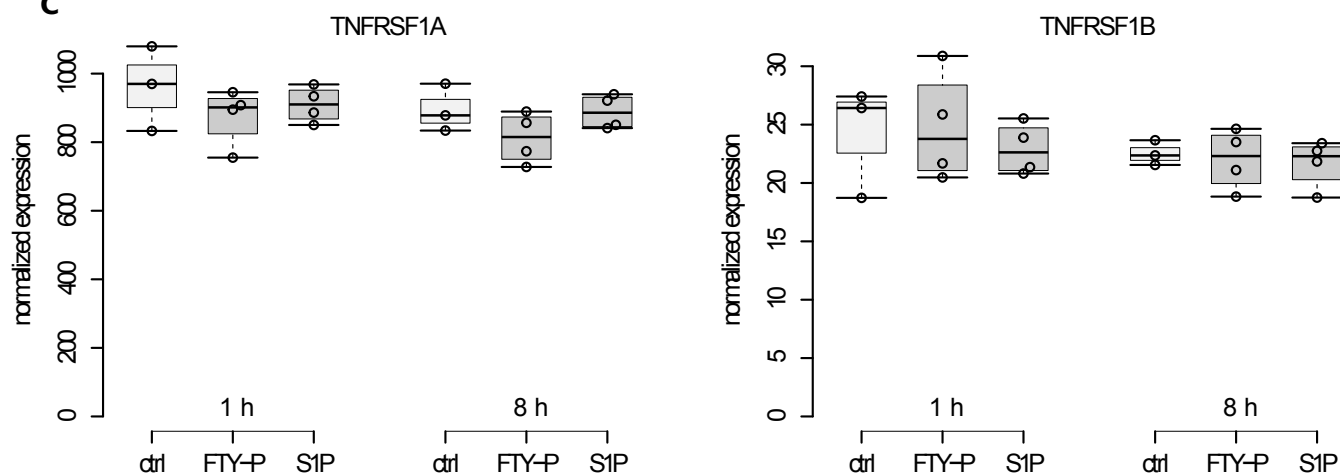

Supplement: Additional file 5: Figure S3. — Analysis of possible interactions between FTY-P and TNF. (A) Human U373 astrocytoma cells were stimulated with FTY-P (1 μM) or S1P (1 μM), followed after 1 h by stimulation with TNF (0.025 μg/ml) when indicated. Eight hours later, cell lysates were harvested and expression of SPHK1, SPHK2, SGPL1, and SGPP1 was determined by quantitative PCR (mean ± SEM of five independent biological replicates; two-tailed paired t tests). (B) Human U373 astrocytoma cells were transfected with a luciferase-based NFκB-reporter and stimulated with FTY-P (1 μM) and TNF (0.025 μg/ml) 1 h later. Eight hours later, NFκB-activation was determined (mean ± SEM of combined data of 8–11 independent biological replicates). (C) FTY-P and S1P do not increase TNF receptor expression. Human primary astrocytes were treated with FTY-P, S1P, or vehicle control for 1 or 8 h (see Fig. 1, same microarray experiment). Normalized gene expression for TNFRSF1A (the main receptor for soluble TNF) and TNFRSF1B is displayed on the Y axis. Boxplots indicate median and first/third quartile, with whiskers extending to outliers up to 1.5 × interquartile range. [file 12974_2015_393_MOESM5_ESM.pdf]

Suppl. Fig. 4

**A**

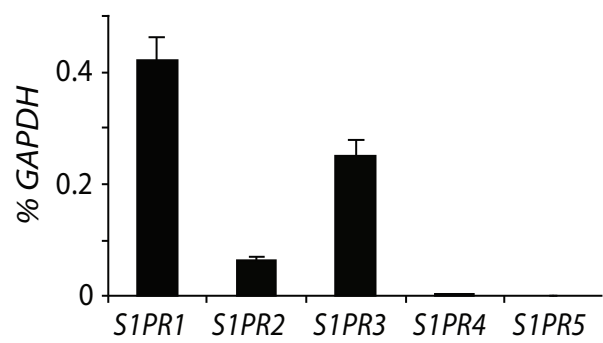

**B**

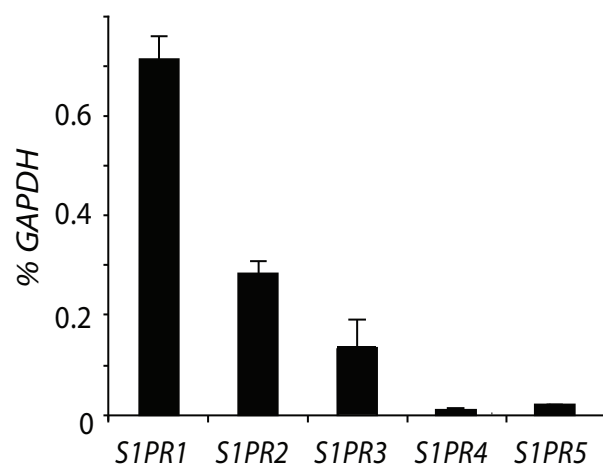

Supplement: Additional file 6: Figure S4. — Human primary astrocytes and U373 astrocytoma cells mainly express S1P receptor types 1 and 3. Expression of S1PR1-5 was determined in human primary astrocytes (A) and human U373 astrocytoma cells (B) by qPCR (mean ± SD of two technical replicates). [file 12974_2015_393_MOESM6_ESM.pdf]
