# Supplementary material for: Fingolimod induces neuroprotective factors in human astrocytes
Source: J Neuroinflammation. 2015 Sep 30;12:184. doi: 10.1186/s12974-015-0393-6 (PMC4589103; doi:10.1186/s12974-015-0393-6)
Supplement: Additional file 2: Table S1. — siRNA sequences. Sequences of the sense strand of siRNAs targeting S1P1 and S1P3 are listed. All siRNAs are Silencer® Select Validated siRNAs (Life Technologies). [file 12974_2015_393_MOESM2_ESM.docx]

**Table S1: siRNA sequences**

| **siRNA name** | **sequence** |
| --- | --- |
| S1P1 (1) | 5´-GCAGCUCGGUCUCUGACUAtt-3´ |
| S1P1 (2) | 5´-GCACCACGGUCUUCACUCUtt -3´ |
| S1P3 (1) | 5´-GGAUGCUUGUAUAUUCGUAtt-3´ |
| S1P3 (2) | 5´-GCACUUGACAAUGAUCAAAtt -3´ |

Sequences of the sense strand of siRNAs targeting S1P1 and S1P3 are listed. All siRNAs are Silencer ® Select Validated siRNAs.
